# Improving detection of impacted animal bones on lateral neck radiograph using a deep learning artificial intelligence algorithm

**DOI:** 10.1186/s13244-023-01385-x

**Published:** 2023-03-16

**Authors:** Yueh-Sheng Chen, Sheng-Dean Luo, Chi-Hsun Lee, Jian-Feng Lin, Te-Yen Lin, Sheung-Fat Ko, Chiun-Chieh Yu, Pi-Ling Chiang, Cheng-Kang Wang, I.-Min Chiu, Yii-Ting Huang, Yi-Fan Tai, Po-Teng Chiang, Wei-Che Lin

**Affiliations:** 1 Department of Diagnostic Radiology, Kaohsiung Chang Gung Memorial Hospital, Chang Gung University College of Medicine, 123 Ta-Pei Road, Niao-Sung, Kaohsiung, 83305 Taiwan; 2 Department of Otolaryngology, Kaohsiung Chang Gung Memorial Hospital, Kaohsiung, Taiwan; 3 Next E-Commerce Technology Co., LTD., Taichung, Taiwan; 4 Department of Emergency Medicine, Kaohsiung Chang Gung Memorial Hospital, Kaohsiung, Taiwan; 5 Department of Radiology, Jen Ai Chang Gung Health, Dali Branch, Taichung, Taiwan

**Keywords:** Artificial intelligence, Retrospective studies, Lateral neck radiograph, Animal bone impaction

## Abstract

**Objective:**

We aimed to develop a deep learning artificial intelligence (AI) algorithm to detect impacted animal bones on lateral neck radiographs and to assess its effectiveness for improving the interpretation of lateral neck radiographs.

**Methods:**

Lateral neck radiographs were retrospectively collected for patients with animal bone impaction between January 2010 and March 2020. Radiographs were then separated into training, validation, and testing sets. A total of 1733 lateral neck radiographs were used to develop the deep learning algorithm. The testing set was assessed for the stand-alone deep learning AI algorithm and for human readers (radiologists, radiology residents, emergency physicians, ENT physicians) with and without the aid of the AI algorithm. Another radiograph cohort, collected from April 1, 2020, to June 30, 2020, was analyzed to simulate clinical application by comparing the deep learning AI algorithm with radiologists’ reports.

**Results:**

In the testing set, the sensitivity, specificity, and accuracy of the AI model were 96%, 90%, and 93% respectively. Among the human readers, all physicians of different subspecialties achieved a higher accuracy with AI-assisted reading than without. In the simulation set, among the 20 cases positive for animal bones, the AI model accurately identified 3 more cases than the radiologists’ reports.

**Conclusion:**

Our deep learning AI model demonstrated a higher sensitivity for detection of animal bone impaction on lateral neck radiographs without an increased false positive rate. The application of this model in a clinical setting may effectively reduce time to diagnosis, accelerate workflow, and decrease the use of CT.

**Supplementary Information:**

The online version contains supplementary material available at 10.1186/s13244-023-01385-x.

## Key points

- The AI-aided interpretation improved the accuracy for doctors of every specialty.
- The AI model accurately identified more cases than the radiologists’ reports.
- The AI model increased the detection rate and may decrease required CT imaging.

**Supplementary Information:**

The online version contains supplementary material available at 10.1186/s13244-023-01385-x.

## Introduction

Foreign body ingestion and subsequent impaction, such as fish bones impacted in the upper digestive tract, is a common cause of emergency department visits with symptoms ranging from pain and dysphagia to airway compromise [1]. However, patient descriptions of symptoms including a foreign body sensation, dysphagia, or pharyngeal pain are not always sufficiently specific to localize the foreign body [2]. Nonetheless, a detailed recording of patient history and a thorough physical examination remain standard for initial patient management. Although subsequent management may differ across institutions, patients often receive a lateral neck radiograph after a negative oropharyngeal examination [3]. If the result of the lateral neck radiograph is negative, additional tests including a non-enhanced computed tomography scan (NECT) or direct esophagoscopy may then be performed. These tests may incur a high cost and are either more invasive or produce a higher dosage of radiation.

The lateral neck radiograph is a relatively inexpensive and accessible management tool for assessing patients with suspected animal bone impaction in the cervical esophagus. However, previous studies have indeed reported varying detection rates [4–6], possibly due to variable interpretation skills among doctors and different radio-opacities presented by different foreign bodies [3, 7]. In addition, the accurate interpretation of the lateral neck radiograph may be affected by the specific subspecialty and related experience of the image interpreter [8].

In recent years, the application of artificial intelligence (AI) techniques, specifically deep learning algorithms, have been investigated in the field of radiology to enhance the diagnostic process, accuracy rates, lesion detection, and prognostic prediction models [9]. The use of AI can greatly improve the workflow of radiologists for labor-intense tasks such as lesion detection and segmentation [10]. Other advantages include improved diagnostic accuracy, automation of tasks such as image segmentation, and the potential to reduce healthcare costs through improved diagnostic efficiency and the reduced need for additional imaging studies [11–13]. However, there are several challenges related to the development and use of AI in medical imaging. One of the primary challenges is the limited availability of high-quality, labeled medical imaging data which could be further aggravated by concerns for privacy and data protection. There are also concerns regarding the interpretability of AI due to the difficulty in understanding the decision-making processes of AI algorithms [14]. Additionally, the formulation of guidelines and policies regulating the use of AI in clinical practice lags the development of AI applications [11]. Currently, developing AI algorithms for foreign body detection on plain radiography lacks large labeled open databases and few studies have reported promising results. A recent study reported on the high accuracy achieved by an AI model in the detection of swallowed batteries and coins from a relatively small training sample [15]. However, since animal bones have a relatively lower radio-opacity and present with variable shapes and sizes, the detection rate on radiograph is much lower than that for metallic objects [6, 16]. In this study, we aimed to develop a deep learning algorithm for the automatic identification and labeling of impacted animal bones on lateral neck radiograph. In addition to the conventional method for validating the algorithm in a separate test set, we analyzed the efficacy of the deep learning AI model in a simulated clinical setting with a clinical cohort from a different time period.

## Materials and methods

### Study population

**Fig. 1:**
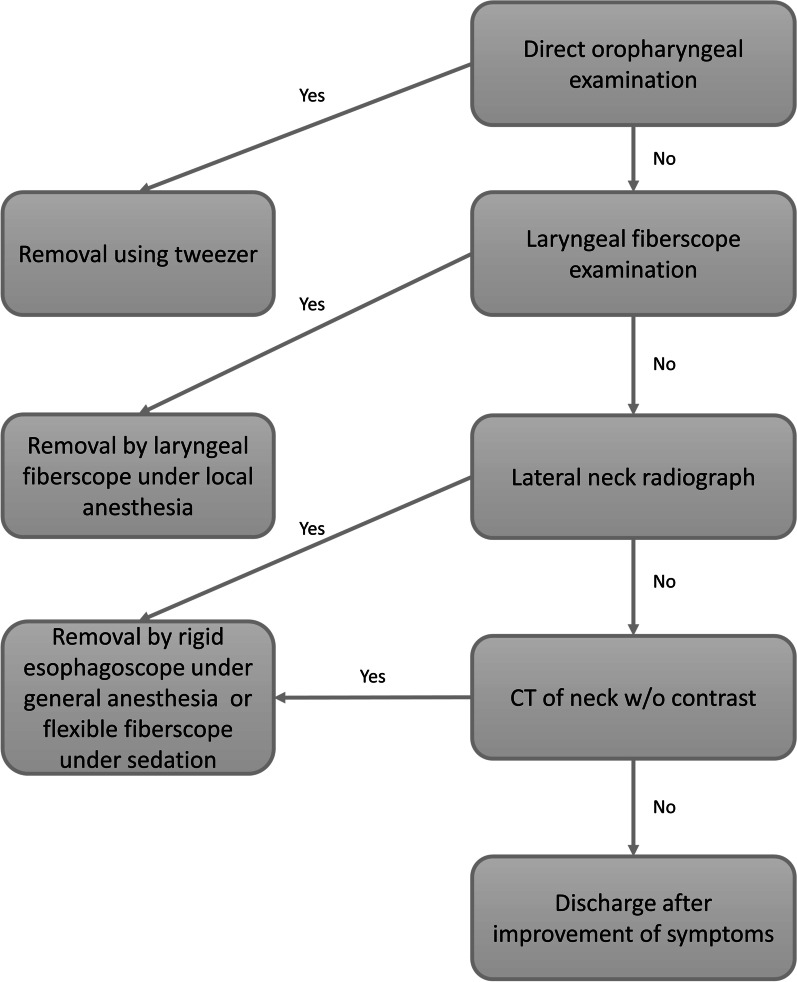
Clinical workflow for suspected animal bone impaction. Patients presenting with animal bone impaction will receive direct oral examination followed by laryngeal fiberscope if the oral examination is not revealing. Any bones found will be directly removed during the examination. If no foreign body is detected, then a lateral neck radiograph will be performed. If the foreign body is still not identified, then a CT without contrast medium administration from the tongue base to the stomach will be performed. If the foreign body is detected in the esophagus, either by plain radiograph or CT, then the foreign body will be removed by rigid esophagoscope under general anesthesia or flexible fiberscope under sedation. Bones detected on lateral neck radiograph will exempt the patient from further CT studies unless there are concerns for complication. If no foreign body is detected on CT, then the patient will be discharged if symptoms improve

We retrospectively reviewed all patients presenting to the emergency department of our hospital with the chief complaint of ingested foreign body from January 2010 to March 2020. The patient enrollment process began by searching through medical health records for patients presented to the emergency department with a diagnosis of foreign body ingestion which received either rigid esophagoscope or flexible fiberscope. After the initial search, the medical records and procedure notes were reviewed to identify eligible cases for imaging review. Cases with an alternative diagnosis or those with foreign body ingestion other than animal bones were excluded. After which, every lateral neck radiograph was reviewed with reference to procedure notes, photograph of the specimen, and CT if available (Additional file 1: Fig. S1). All patient cases were confirmed under flexible fiberscope or rigid esophagoscope. There was no age restriction, although we excluded pediatric patients with coin or battery ingestion as their clinical presentations are distinct and often very apparent on lateral neck or frontal chest radiograph. Cases of impaction with food bolus, plant seed, metal denture, and plastic materials were also excluded as there were too few cases available and thus not suitable for the development of the AI model. Our hospital’s Institutional Review Board approved this study. The clinical workup for patients presenting with suspected animal bone impaction in our hospital is similar to a previous report [17] and is shown in Fig. 1.

### Image annotation and preprocessing

The routine exposure settings used for lateral neck radiography in our hospital were adjusted based on the patient’s body type and age. For teenagers and adults, we used an exposure setting of 63 kVp and 16 mAs, while for children and babies, the setting was 60 kVp and 12 mAs. For obese patients with a short neck, we used an exposure setting of 66 kVp and 20 mAs. All images were labeled using a bounding box containing the fishbone. The identification of the animal bone was performed retrospectively, based on the endoscope report, a photograph of the specimen, and CT image if available. Cases with unidentifiable animal bones after referencing to these available data were excluded from the study. A total of 1783 lateral neck radiographs were included and labeled. The input data files were DICOM files, with the image data extracted as pixel arrays. The pixel data were then applied with contrast adjustment to improve object visibility and enhanced with edge sharpness to improve image clarity. For image training purposes, pixel data were also scaled to a floating-point digit type of 0 to 1 from the original 16-bit data.

### Development of the deep learning algorithm

Our model is based on the Faster-RCNN convolution neural network with Inception-resnet-v2 architecture pre-trained model, where Faster-RCNN provides a region-based convolution network and the Inception-resnet-V2 provides feature extraction from the intended region. The network inputs were bounding box coordinates representing the ingested animal bone and trained with a single label. The output was the bounding box coordinate of the predicted ingested bones. The training and testing environments were implemented on TensorFlow 2.5 and processed with a discrete graphics card.

The data included 1783 DICOM files consisting of lateral neck radiograph images, fifty of which were randomly selected to construct the testing set. The remaining 1733 files were split into training and validation datasets, at a ratio of approximately 6:4 (1213:520). The Adam optimizer was configured as the training optimizer, wherein the learning rate was set to 0.0001 with a training batch size of 1. As there was only a single label in the training process, no class balance process was conducted. The training was set to compute approximately 200 epochs. The correct detection for each case was defined by the intersection over union (IoU) between the labeled bounding box and the output bounding box. An IoU > 0.7 was defined as correct. The model was trained on a custom workstation with an Intel Core i9-9900 K CPU, 64 GB of DDR4 RAM, 1 TB SSD, 2 TB hard drive, and one RTX 2070 GPU.

### Evaluating the deep learning algorithm

#### The testing set

A total of 50 lateral neck radiographs with animal bones and 50 without animal bones were randomly combined as the test set. The human readers included 2 radiologists, 2 radiology residents, 2 ER physicians (1 board-certified and 1 in-training physician), and 2 ENT physicians (1 board-certified and 1 in-training physician). The sensitivity, specificity, and accuracy were evaluated for both the AI algorithm and the human readers. All participants involved in the test were unaware of the percentage of subjects with a positive foreign body impaction in the test set, their performance on the test, and of the correct answer to each reading. All participants retook the examination with the aid of the AI model after a washout period of 6 months to assess the performance of the AI-assisted interpretation.

#### Simulating application of the deep learning algorithm in a real-world clinical setting

From April 1, 2020, to June 30, 2020, a total of 209 patients with a chief complaint of animal bone impaction presented to the ER were included in the study. Of these, 83 were identified and removed by direct oral examination or laryngeal fiberscope, and 20 were positive for bone impaction in the esophagus, while the rest 106 were negative for bone impaction. The 126 patients (20 positives and 106 negatives for impacted bones in the esophagus) were included in the analysis. All 126 patients received lateral neck radiograph examinations. The AI performance was compared with the reports of radiologists.

### Statistical analyses

In this study, continuous variables (age) and categorical variables (sex) were analyzed using ANOVA and chi-squared tests. The results are reported as mean ± standard deviation (SD). All demographic data analyses were conducted using Statistical Product and Service Solutions (SPSS) software version 19 for Windows (IBM). *p* values < 0.05 were considered statistically significant.

## Results

### Datasets

The demographic data of patients included in the training, validation, and testing datasets are shown in Table 1. There were 1783 lateral neck radiography with identifiable bone impactions from January 2010 to March 2020. Among the 1783 lateral neck radiographs, 50 were randomly selected as the testing dataset. The remaining 1733 radiographs were split into the training dataset (1213) and validation dataset (520). The patients in the training and validation sets were on average older than those in the testing and simulation sets, while there was no difference in the sex. The images included in the formation of the datasets were generated from 3 different manufacturers: CARESTREAM, SHIMADZU, and TOSHIBA.

**Table 1:** Demographic data of patients included in the training, validation, testing, and simulation datasets

	Training set	Validation set	Testing set	Simulation set	*p*
Number of patients	1213	520	100	126	
Age (year)	56.27 ± 16.87	55.12 ± 18.56	47.44 ± 23.06	45.18 ± 23.93	< 0.001*
Sex (M, F)	484, 729	208, 312	41, 59	61, 65	0.319
*Manufacturer/model*					
CARESTREAM/DRX-evolution	610	294	71	58	
SHIMADZU/RADspeed Pro	15	8	1	42	
TOSHIBA/KXO-80G	588	218	28	0	

### Performance of the AI model and doctors

**Fig. 2:**
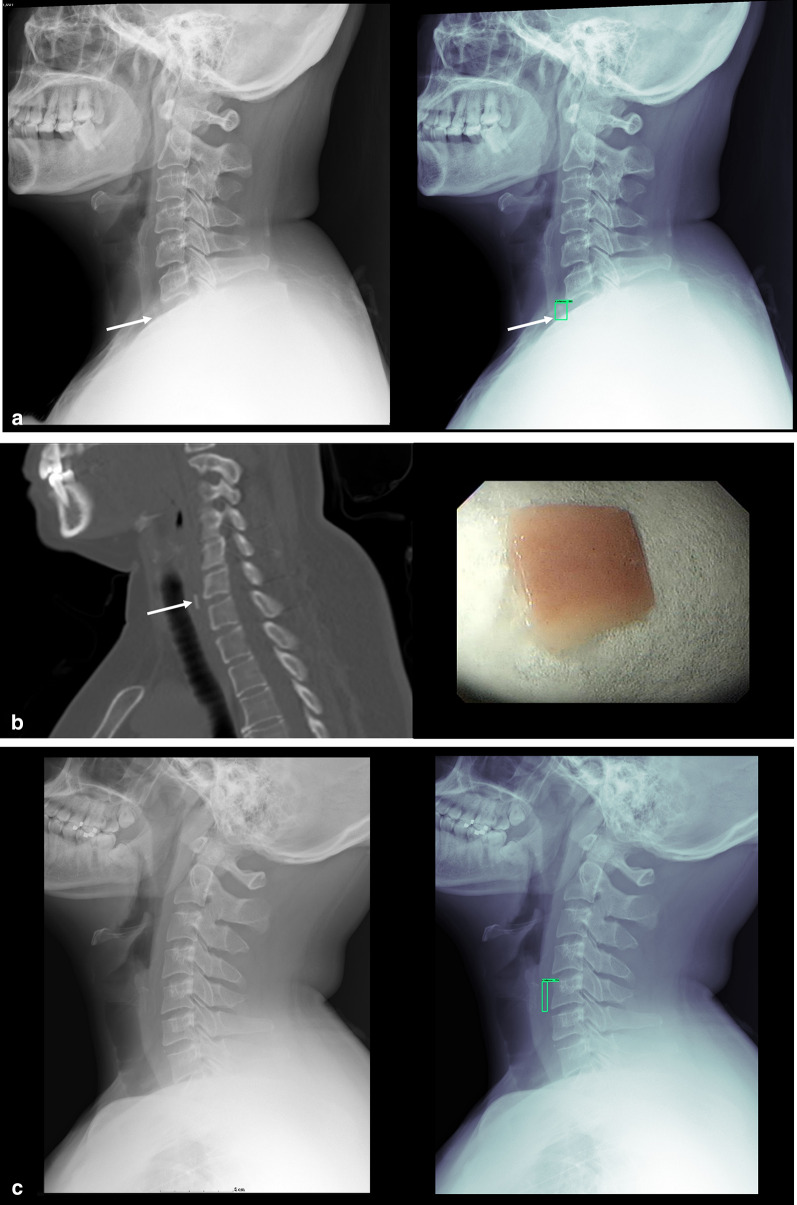
Representative cases of true positive and false positive of the deep learning algorithm. True positive and false positive of the deep learning algorithm. **a** Plain radiograph of the lateral neck and the results of the deep learning algorithm showed a foreign body (arrows) over prevertebral soft tissue at C7 level partially obscured by the shoulder. **b** CT without contrast showed a foreign body over the cervical esophagus at C7 level and the photograph of the specimen retrieved revealed a flat rectangular shaped fish bone. **c** The deep learning algorithm misinterpreted the calcified postero-inferior lamina of the thyroid cartilage as a foreign body. This can be confidently recognized by identifying the two parallel lines conforming to the shape of the bilateral postero-inferior lamina of the thyroid cartilage

**Fig. 3:**
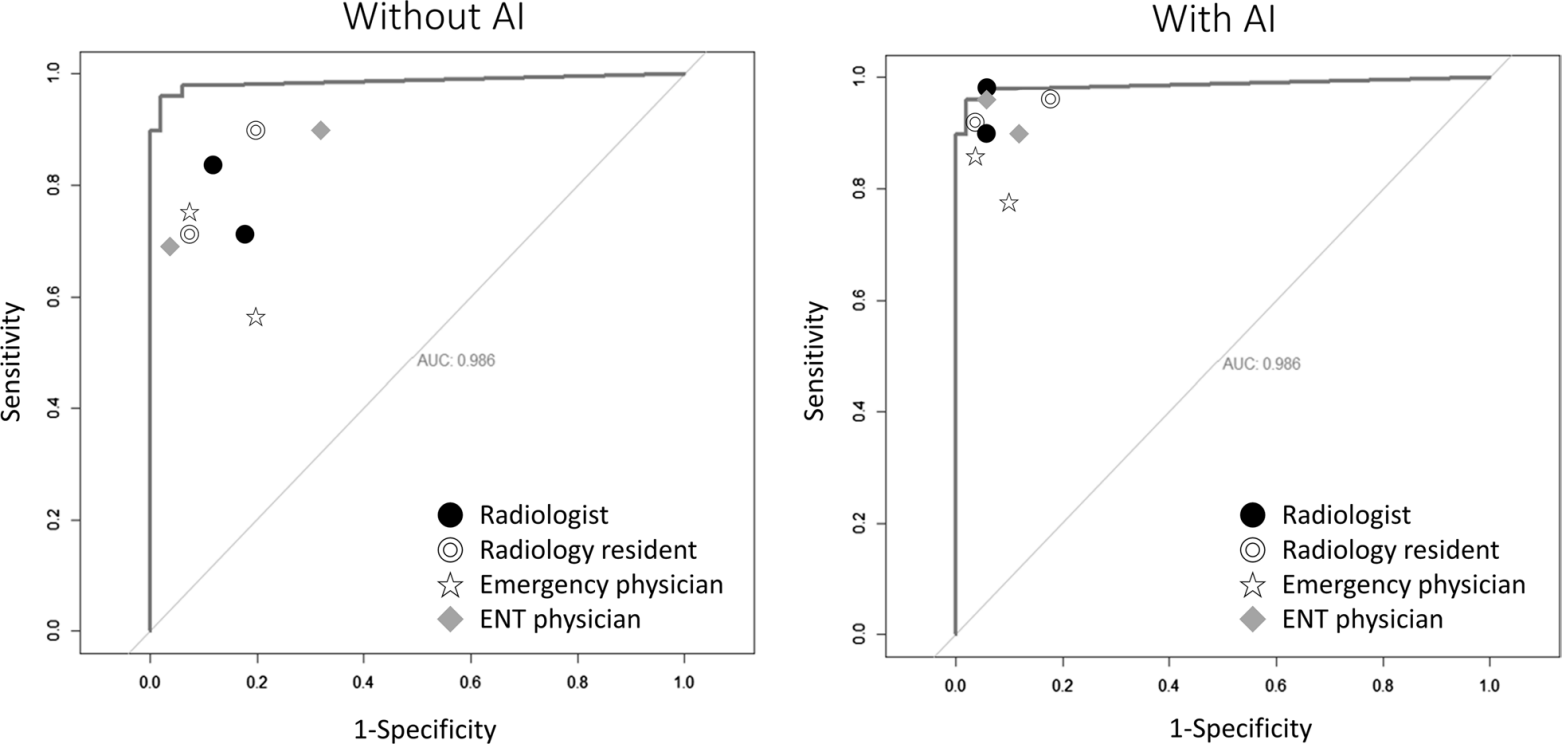
Physicians’ performances with and without AI assistance. ROC curve showing the performance of each physician with and without AI assistance. The two readings were separated with a washout period of 6 months

Representative cases from the true positives and false positives from the deep learning algorithm are shown in Fig. 2. The respective performances of the AI model and doctors on the testing dataset comprised of 100 patients are shown in Table 2. The sensitivity, specificity, and accuracy of the model were 96%, 90%, and 93% respectively. Among the doctors, radiologists demonstrated the highest sensitivity and accuracy. After a washout period of 6 months, all doctors exhibited improved performance with the aid of the AI model, with an accuracy improvement of more than 10% in doctors of every specialty, as shown in Table 2 and Fig. 3.

**Table 2:** Performances on the testing set of the AI model and doctors with and without AI assistance

	Sensitivity (%)	Specificity (%)	Accuracy (%)
AI performance	96	90	93
*Doctor alone performance*			
ER physician	67	86	76.5
ENT physician	70	82	76
Radiologist	82	83	81.5
Radiology resident	76	82	79
*Doctor with AI-aided performance*			
ER physician	82	93	87.5
ENT physician	93	91	92
Radiologist	94	94	94
Radiology resident	92	96	94

### Simulation of real-world use in a clinical setting

Within a timeframe of 3 month, a total of 126 patients with a chief complaint of animal bone impaction presented to the ER with negative oral and laryngeal fiberscope examination were analyzed. Of these, 20 were positive for bone impaction in the esophagus, while the rest 106 were negative for bone impaction. The results of the AI model and radiologists’ reports are shown in Table 3. Among the 106 negative cases, 71 were discharged with follow-up at the ENT outpatient department, while 35 patients received a CT scan from the base of the tongue to the stomach due to persistent symptoms. Among the 20 cases who were found to be positive for animal bone impaction, 11 were recorded as negative in the radiologists’ lateral neck radiograph reports. Each of those 11 cases received a subsequent CT scan, on which impacted bones were indeed identified and then removed by rigid endoscope under general anesthesia. After referencing the CT imaging, 5 of the 11 cases were retrospectively found to be identifiable on the original radiograph. The 6 cases which were retrospectively deemed not visible on the original lateral neck radiograph were cases in which the impacted bone was either obscured by the shoulder or out of field (2 at C7 level; 2 at T1 level; 2 at T3 level). Our AI model correctly identified 12 of the 20 cases with animal bone impaction, including 3 cases which were missed in addition to the 9 cases identified in the radiologists’ reports. As expected, the 6 cases which were obscured by the shoulder or out of field were also missed by the AI model. Among the 106 cases without animal bone impaction, 10 cases were misinterpreted as positive in the radiologists’ reports, while the AI model misinterpreted 7 cases as positive. Among these false positive cases, only 2 overlapping cases existed between the AI model and radiologists’ reports.

**Table 3:** Performance on the simulation set of the AI model and the radiologists’ reports

	Sensitivity	Specificity	Accuracy (%)
Deep learning AI model	60% (12/20)	93% (99/106)	88
Radiologist’s reports	45% (9/20)	91% (96/106)	83

## Discussion

We herein present a deep learning artificial intelligence algorithm for the detection of impacted animal bones on lateral neck radiography. The algorithm demonstrated a non-inferior detection rate as compared to human readers in the testing set. We further investigated the potential application of this algorithm in a real-world clinical setting with a simulation set consisting of patients enrolled during a different time period and evaluated in a direct comparison with radiologists’ reports. The direct comparison revealed that the deep learning algorithm correctly identified 3 more animal bones than the radiologists on lateral neck radiographs.

Several previous studies have investigated the value of lateral neck radiography in patients with impacted foreign bodies, with reported sensitivities ranging from 10% to more than 90% [4–6]. The diverse sensitivities may be attributed to different components of the foreign bodies and their locations. Of note, studies having reported a higher sensitivity did not distinguish cases according to the specific type of foreign body [4, 5, 18], whereas those that reported a lower sensitivity included only impacted animal bones [6, 19, 20]. For animal bone impaction, studies have indeed suggested that plain radiograph is of little value, while CT demonstrates the highest accuracy [19, 20]. Meanwhile, more recent studies have suggested that a lateral neck radiograph be performed only after a negative laryngeal fiberscope examination, as this examination is well-tolerated for patients and the detection rate of lateral neck radiograph for animal bones located in the oropharynx is poor [6, 18]. In our institution, all patients presenting with suspected foreign body impaction will initially receive a laryngeal fiberscope examination. Therefore, the main value of a lateral neck radiograph is to detect foreign bodies that are inaccessible by the laryngeal fiberscope, while impacted bones detected on the lateral neck radiograph will exempt the patient from a further CT scan. The positive identification of impacted animal bones on plain radiograph will effectively act to accelerate the diagnostic and management processes while decreasing the radiation dosage and medical fee.

Radiographic signs for impacted animal bones on lateral neck radiography include direct visualization of radiopaque density and indirect signs, including presence of abnormal air column lucency, loss of cervical lordosis, and increased prevertebral soft tissue thickness [21]. However, since the indirect signs may merely reflect local soft tissue irritation [21, 22], unless the animal bone is directly visualized, further study, such as NECT, is often performed before a definitive treatment can be determined. Similar to a previous report [8], the interpretation accuracy of lateral neck radiography for doctors with different years of experience and subspecialties varies in this study. Although with the aid of the deep learning AI algorithm, every doctor exhibited improved accuracy. The ability of an interpreter to accurately identify animal bones of various sizes and in variable locations on lateral neck radiograph gradually improves with experience, thus our deep learning AI model may effectively act to accelerate and enhance this acquired ability.

There was a decrease of more than 30% in sensitivity between the test set and the simulation set, for both the deep learning AI model and radiologists. The main explanation for this result was likely the different cohorts comprising the two sets. More specifically, the test set, in addition to the training and validation sets, included only cases in which the animal bones were identifiable on lateral neck radiography. By contrast, the simulation set had no such exclusion criteria, and thus included cases which would have been excluded from the testing set. Therefore, the decreased detection rate observed in the simulation set was potentially due to the intrinsic limited effectiveness of the lateral neck radiograph to detect impacted animal bones.

Included in the simulation set were 14 cases with animal bones which were retrospectively deemed as identifiable on lateral neck radiography, with or without reference to CT imagery. Among these 14 cases, 5 were missed by the radiologists and received subsequent CT scans. Our deep learning model accurately detected 3 more cases as compared to the radiologists, which would translate into 3 fewer CT scans performed if the model was applied in clinical practice. Furthermore, as most of the false positives made by the AI model and the radiologists did not overlap, the AI model could act to complement the interpretation of the lateral neck radiograph, thereby achieving a lower false positive rate.

The radiographic evaluation of patients with animal bone impaction varies across institutions, with plain radiograph being the first-line radiological investigation [17, 23] to completely abandoning plain radiograph in the evaluation process [24, 25]. Although lacking sensitivity, a positive result on the plain radiograph is sufficiently specific to warrant direct treatment without the need for further imaging [23]. As many missed cases were retrospectively identifiable, our AI model may enhance the interpretation process of lateral neck radiographs for the detection of animal bone impaction, thereby decreasing the need for further imaging and accelerating the clinical workflow. However, the radiograph interpreter should be aware of the factors which may affect the interpretation of lateral neck radiograph. In clinical practice, it is often challenging to interpret lateral neck radiographs in older patients due to complex calcification and ossification structures in the neck which can obscure the image or be mistaken for swallowed animal bones. In this study, no animal bones were missed by the AI algorithm among pediatric patients. This could be attributed to better soft tissue penetration with no obscuring calcification or ossification structures in the neck. However, since pediatric patients only made up a small proportion of the samples (3 cases out of 50 positive cases in the testing data set, and 2 cases out of 20 positive cases in the simulation data set), further studies involving larger numbers of pediatric patients are needed to reach more definitive conclusions.

One of the main challenges in the integration of AI in radiological practice is the need for radiologists to be trained in the use of AI algorithms and to understand the decision-making processes of the AI models [26]. Another challenge is the need for collaboration between radiologists and AI developers to ensure that the AI algorithms are properly validated and the results are properly interpreted [27]. Lastly, the integration of AI in radiological practice also requires the development of infrastructure and the integration with the existing Radiology Information System (RIS) and Picture Archiving and Communication System (PACS) in the hospital. The AI model in this study is a relatively straightforward application aimed at a very specific clinical scenario for which the training of its use would be simple and fast. However, users must note that the algorithm was trained in a single institution, such that the accuracy of the model may be affected by distinct varieties of ingested animal bones in cultures with different diets.

The strength of this study lies in the fact that the labeling and classification was not based on radiologists’ reports, but rather retrospectively referenced to the CT, endoscopy, and photograph of the specimen to ensure the quality of the data used for developing the algorithm. Meanwhile, there are indeed several limitations. First, the data used to train the model were from a single institution. Although the data were attained by different brands and models of x-ray machines in a time period of 10 years, external validation is still needed for further verification. Since not only the brand and model of x-ray machines may affect the final results, animal bones from different species of animals (particularly different species of fish) may also impact detection rates [20, 28]. Therefore, the results may vary in different geographic zones with different diets. Second, the simulation section of the study was conducted in a relatively short period of time, while clinical efficacy may be better evaluated by a prospective clinical trial. Third, although our results demonstrate the potential benefits of AI-assisted detection on plain radiograph to decrease the need for CT imaging, the detection rate is limited by the intrinsic limitations of plain radiography, particularly for bones impacted in the thoracic esophagus. Lastly, the deep learning AI model was trained to specifically identify animal bones on lateral neck radiographs and is not intended to replace a formal radiological report. Rather, the purpose of this AI model is to assist the interpreter to quickly identify impacted animal bones on lateral neck radiograph, while the interpreter should still scrutinize the imagery for other potentially critical findings, such as abnormalities of the cervical spine, airway, or other soft tissue lesions of the neck.

In conclusion, our deep learning AI model demonstrates a superior sensitivity for the identification of impacted animal bones on lateral neck radiograph without an increased false positive rate. The application of our AI model in clinical practice may accelerate the diagnostic process, thereby improving workflow and decreasing the need for CT imagery.

## Supplementary Information

**Additional file 1**.** Supplementary Fig. 1**. Flow chart for patient enrollment.

## Data Availability

The datasets generated or analyzed during the study are available from the corresponding author on reasonable request.

## Abbreviations

AI: Artificial intelligence
IoU: Intersection over union
NECT: Non-enhanced computed tomography scan
