# Supplementary material for: Improving detection of impacted animal bones on lateral neck radiograph using a deep learning artificial intelligence algorithm
Source: Insights Imaging. 2023 Mar 16;14:43. doi: 10.1186/s13244-023-01385-x (PMC10020388; doi:10.1186/s13244-023-01385-x)

ELECTRONIC SUPPLEMENTARY MATERIAL

Improving detection of impacted animal bones on lateral neck radiograph using a deep learning artificial intelligence algorithm

Supplementary Fig. 1

Flow chart for patient enrollment

Searching through the medical health records for patients presented to the emergency department with a diagnosis of foreign body ingestion that received either rigid esophagoscope or flexible fiberscope yielded 3,935 potentially eligible patients. After reviewing the medical records and procedure notes, 2,574 subjects were included for imaging review. After which, every lateral neck radiograph was reviewed with reference to procedure notes, photo of the specimen, and CT if available. This process excluded 791 lateral neck radiographs due to non-visibility of the animal bones. The remaining 1,783 lateral neck radiographs were then included for image annotation.

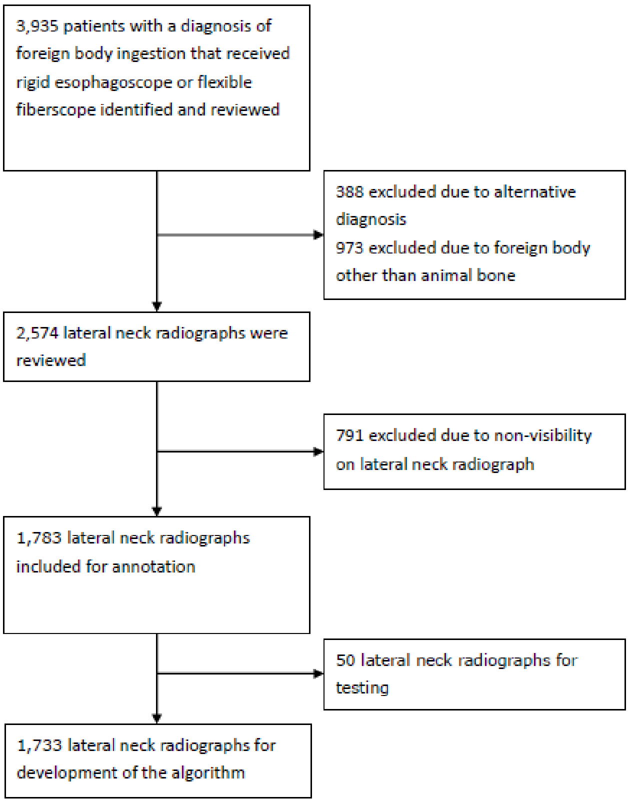

Supplement: Supplementary file 1 — Additional file 1. Supplementary Fig. 1. Flow chart for patient enrollment. [file 13244_2023_1385_MOESM1_ESM.pdf]
